# Expression of Estrogen Receptors (ER), Progesterone Receptors (PR) and HER-2/neu receptors in Endometrial Carcinoma and their associations with histological types, grades and stages of the tumor

**DOI:** 10.12669/pjms.342.13637

**Published:** 2018

**Authors:** Samina Waqar, Saleem Ahmad Khan, Tariq Sarfraz, Saba Waqar

**Affiliations:** 1 Dr. Samina Waqar, M.Phil. Department of Histopathology, Army Medical College/ Military Hospital, Rawalpindi, Pakistan; 2 Dr. Saleem Ahmad Khan, PhD. Department of Hematology, Army Medical College/ Military Hospital, Rawalpindi, Pakistan; 3 Dr. Tariq Sarfraz, FRC Path. Department of Histopathology, Army Medical College/ Military Hospital, Rawalpindi, Pakistan; 4 Dr. Saba Waqar, M. Phil. Department of Dental Material, Army Medical College/ Military Hospital, Rawalpindi, Pakistan

**Keywords:** Endometrial Carcinoma, Estrogen Receptors, Her-2/Neu, Progesterone Receptors

## Abstract

**Objective:**

To study and detect immunohistochemical expression of Estrogen Receptors, Progestrone Receptors and HER-2/neu Receptors in Endometrial Carcinoma (EC) and to find their associations with histological types, grades and stages of the tumor.

**Methods:**

A cross sectional study of one year duration from January 2016 to January 2017 was conducted at Histopathology department of Army Medical College, Rawalpindi. A non-probability purposive sampling technique was used to include 56 cases of EC. The specimens were tested for ER, PR and HER-2/neu expression using immunohistochemical analysis. Data was analyzed in SPSS and the significance of association of expression of the receptors with histological types, grades and stages of the tumor was assessed.

**Results:**

Significant association of Her-2/neu overexpression with histological types and grades of EC was seen, whereas the association of ER and PR expression with histological types, grades and stage of EC was statistically insignificant.

**Conclusion:**

It is suggested that EC showing over expression of HER2/neu with immunohistochemistry may be treated with anti HER-2/neu treatment with better chances of survival and decreased post-treatment morbidity.

## INTRODUCTION

Endometrial Carcinoma (EC) is the fourth most common cancer in women. About 90% of these tumors are sporadic and 10% hereditary.^1^ According to American Society of Cancer (ASC) 54,870 new cases of EC emerged and 10,170 deaths occurred in 2015 in USA.^2^ In Pakistan EC is causing up to 3.1% of female malignancies.^3^ In India, the rates are 4.3 per 100,000.^4^ Despite of these facts the general population is not aware of EC.^2^ The increased use of estrogen products reflected by their increased sale in 1960s and 1970s till 1975 for the treatment of post menopausal symptoms may have resulted in increased emergence of endometrial cancer.

The two types of EC, Type-1 and Type-2 which are different in their etiology, clinical behavior and treatment modalities were originally described by Bokhman JV et al. in 1983.^5^ There was emergence of another group that is mixture of these two. Still another group has carcinomas like Carcinosarcoma which are high grade and poorly differentiated. In this era of specific targeted therapy for cancers, search for biological markers is an ongoing effort in various types of cancers causing high mortality.

The role of hormonal treatment and targeted therapy against HER-2/neu with Herceptine is already being practiced for breast carcinoma, and gastric carcinomas including gastro-esophageal adenocarcinomas.^6^ Specific target therapy for these tumors are in practice now^7^ and response of targeted therapy for these tumors depends on their expression of HER 2/neu.^8^

Very aggressive endometrial carcinomas including high grade serous carcinoma are found to be less or non-responsive to usual chemotherapy.^9^ One study shows that serous carcinoma forms 10% of all EC.^10^ Some of these cases have shown significantly positive results with better survival when treated with Transtuzumab^11^, making targeted therapy more desirable.

Endometrial carcinoma having high incidence and prevalence and increasing death rates world over motivates one to investigate and search for targeted treatment modalities as surgery and chemotherapy have significant morbidity.

The rationale of the study is to detect the presence of ER, PR and HER-2/neu in EC and any association of immunohistochemical expressions with the histological type, grade and stage of the tumor has been analyzed and highlighted.

## METHODS

The WHO calculator was used to calculate the sample size (confidence level = 95%). The anticipated population proportion was 0.043%, absolute precision required was 5%, and the sample size calculated was 50.^3^ Therefore, 56 cases of EC were included in the study with non-probability purposive sampling.All cases of endometrial biopsies followed by hysterectomy specimens were included, whereas autolyzed samples and endometrial biopsies without hysterectomy specimens were excluded.

Cases of endometrial carcinomas were selected from the surgical samples received from Department of Gynaecology & Obstetrics, Military Hospital (MH) and Combined Military Hospital (CMH) Rawalpindi. Clinical details collected included age, presenting symptoms, comorbid conditions (diabetes, hypertension etc), occupation and other accompanied malignancies. The hysterectomy specimens were collected from operation theatre in 10% formalin. After fixation, proper representative sections were taken. The tissues were enclosed in properly labeled plastic tissue cassettes with perforated walls. The tissue sections processing was done in automatic tissue processor (LEICA TP 1020 Germany) for tissue dehydration, clearing and impregnation with wax. Paraffin blocks were made with the help of paraffin embedding center (LEICA EG 1160 Germany). The sections were cut 3-5 micrometer thick with rotator microtome (LEICA RM 22.5 Germany). Sections from each sample were stained with Hematoxylin-Eosin and Immunohistochemistry was applied.

Immunohistochemical staining with ER, PR and Her-2 was applied on formalin fixed paraffin embedded 3-5 um thick sections. After deparaffinization, antigen retrieval was carried out in citric buffer in microwave for 10 minutes. Then after incubating the tissue in the antibodies, slides were stained with HER-2/ neu, ER, PR. For this BIO SB immunohistochemistry kit was used. Diaminobenzidine was used as chromogenic peroxidase substrate. The slides were counterstained with Hematoxylin. All tests were having a positive and a negative control.

The Hematoxylin and Eosin stained slides and immunohistochemical staining were examined by the M-Phil trainee followed by confirmation by consultant histopathologist (supervisor). Histopathological parameters analyzed, included histological types of tumors, grade and stage of the tumors. Grades and stage of the tumors were analyzed according to WHO and FIGO criteria. Histological types of tumors were decided according to WHO criteria.^12^

The scoring of HER-2/neu was done according to American Society of Clinical Oncology and College of American Pathologists (ASCO/CAP) guidelines.^13^ For the overexpression of the protein HER-2/neu, immunohistochemistry was done on selected representative slides of the tumor. HER-2/neu cases were taken positive when there was complete membranous staining of more than 30% of the tumor cells (Score 3+). The cases with no staining (Score 0) or weak incomplete membrane staining in any proportion or weak complete membranous staining in <10% cells (Score 1+) was considered negative. Incomplete and/or weak/moderate staining within >10% of the cells or complete / circumferential intense staining in <10% cells (Score 2+) was considered as borderline / equivocal. Flourescent In Situ Hybridization (FISH) was recommended for confirmation of equivocal cases.^14^,^15^

## RESULTS

Within the sample for this study 40 (71.4%) patients were aged 50 and more than 50 years (50≥) whereas 16 (28.6%) were less than 50 years (<50) old. Minimum age of the patients was 35 years and maximum age of the patient was 85 years. Mean age was 58.34 years (SD = 9.95).

**Fig.1:**
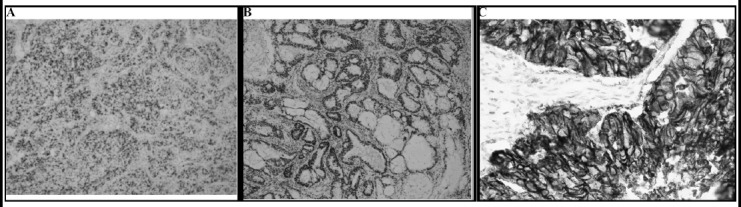
Photomicrograph showing Endometroid carcinoma. **(a)** ER nuclear positivity **(b)** PR nuclear positivity **(c)** Her-2/neu membranous positivity. (What is the Objective power, only high power can show nuclear / cytoplasmic positivity. High power / enhanced resolution pictures required.)

The cumulative results have been given in Table-I. Out of the 56 cases studied, 49 (87.5%) were Endometroid Carcinoma, 3 ((5.36%) were Serous Carcinoma, 2 (3.57%) cases were of Carcinosarcoma, 1 (1.79%) case each of Clear cell Carcinoma and Mucinous Carcinoma. Immuno-histochemical staining (ER, PR, Her-2/neu) on endometrial carcinoma is shown in Fig.1.

**Table-I:** Frequency of ER, PR and Her2Neu receptors and their associations with type, grade and stage of the tumor (n= 56).

Patient Characteristics	Estrogen Receptor			Progesterone Receptor			Her2Neu		

	Positive(%)	Negative (%)	p-value	Positive(%)	Negative(%)	p-value	Positive(%)	Negative(%)	p-value
*Histological Types*									

Endometriod Adenocarcinoma	24(42.85)	25(44.6)	0.351	33(58.9)	16(28.6)	0.604	2(3.6)	47(8.9)	0.002
Serous Ca	0(0)	3(5.4)		2(3.6)	1(1.8)		2(3.6)	1(1.8)	
Clear Cell Ca	0(0)	1(1.8)		0(0)	1(1.8)		0(0)	1(1.8)	
Mucinous Ca	0(0)	1(1.8)		1(1.8)	0(0)		0(0)	1(1.8)	
Carcinosarcoma	1(1.8)	1(1.8)		1(1.8)	1(1.8)		0(0)	2(3.6)	

*Grading*									

I	11((19.6)	17(0.4)	0.222	17(30.4)	11(19.6)	0.538	0(0)	28(50.0)	0.000
II	9(16)	7(12.5)		13(23.2)	3(5.4)		0(0)	16(28.6)	
III	05(8.9)	7(12. 5)		7(12.5)	5(8.9)		4(7.1)	8(14.3)	

*Staging*									

I	19(33.9)	21(37.5)	0.519	27(48.2)	13(23.2)	0.668	3(5.4)	37(66.1)	0.774
II	3(5.4)	5(8.9)		4(7.1)	4(7.1)		0(0)	8(14.3).	
III	2(3.6)	5(8.9)		5(8.9)	2(3.6)		1(1.8)	6(10.7)	
IV	1(1.8)	0(0)		1(1.8)	0(0)		0(0)	1(1.8)	

p≤0.05 considered significant.

Immunohistochemistry revealed 25 (44.6%) ER positive cases and 31(55.4%) ER negative cases. PR positive cases were 37 (66.1%) and PR negative was 19 (33.9%). Her-2 was positive in 4 (7.1%) and negative in 52 (92.9%). The cases that were both ER and PR positive were 27 (48.21%). One triple negative case was Clear cell carcinoma (1.8%)(Table-I).

**Fig.2:**
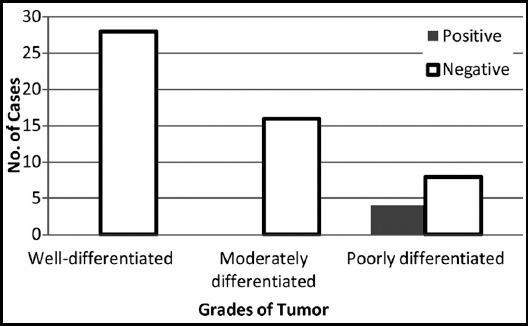
Figure showing association of grades of tumors with HER-2/neu (n=56).

There were 24(42.8%) cases of Endometrioid carcinoma and 1(1.8%) case of Carcinosarcoma positive for ER. There were 33(58.9%) PR positive cases of Endometrioid Carcinoma. Out of three serous carcinomas, 2(3.6%) were PR positive. One (1.8%) each of Mucinous carcinoma and Carcinosarcoma were positive for PR. Clear cell carcinoma was negative for PR.

Her 2 neu staining was done on all 56 cases of endometrial carcinoma, which was positive (Score 3+) in 04 cases (7.14%) and negative (Score 0/1+) in 52 cases (92.85%). No cases with equivocal Her 2 neu staining (Score 2+) was found in any case of this study. Out of 4 Her-2 positive cases, 2(4.1%) were Endometroid carcinoma and 2(66.7%) were Serous carcinoma. Clear cell carcinoma, Mucinous carcinoma and Carcinosarcoma were negative for Her-2.

On applying chi-square test for association between histological types and ER, PR and HER-2/ neu receptors, significant association of Her-2 was seen, with p-value of 0.002. Whereas association of ER and PR were insignificant (p = 0.351 and 0.604, respectively).

According to histological grading 28(50%) were grade I, 16(28.6%) were grade II and 12(21.4%) were Grade III. In grade I, out of 28 cases there were 27 cases of Endometrioid carcinoma and one case of Mucinous carcinoma. In grade II, all 16 cases were of Endometrioid carcinoma. In grade III there were 12 cases out of which 3 cases were of Serous carcinoma, one case of Clear cell carcinoma, two cases of Carcinonosarcoma and six cases of Endometrioid carcinoma. ER positive 11 (19.6%) tumours were in grade I, ER positive grade II tumors were 9(16.9%) whereas in grade III tumors, ER positive were 5(8.9%)(Table-I).

PR positive cases were 17 (30.35%) in grade I, 13(23.21%) in grade II and 7(12.5%) in grade III (Fig.4 and 5). There was no HER-2neu positive case in grade I (0.0%) and grade II (0.0%). All four cases (7.1%) positive for Her-2 receptor were in grade III. Very significant association was seen between grades of EC and Her-2 positive receptors with p value of 0.000., whereas the *p*-values of ER and PR were not significant (0.222 and 0.538 respectively). There were 44(78.6%) cases of Low grade EC (combined Grade-I and grade II) and 12(21.4%) cases of High grade EC (grade III only)(Table-I). ER positive cases in low grade category were 20, PR positive were 30 and none was positive for Her-2. Similarly ER positive cases in high grade category were 5, PR positive were seven and four cases were Her-2 positive. A *p*-value acquired after comparing low grade and high grade carcinomas with different receptors revealed significant results for Her-2 (*p*-value = 0.000) but not for ER and PR (*p*-value = 0.815, 0.523 respectively). Percentage of high grade carcinomas having Her-2 over expression was 25%.

No significant association was seen between stages of EC and type of receptors. The p values of ER, PR and Her-2 were 0.519, 0.668 and 0.774 respectively. There were 40 (71.4%) cases in stage I, 8(14.3%) in stage II, 7(12.5%) in stage III and 1(1.8%) in stage IV..

ER positive cases in stage I were 19(33.9%), 3(5.4%) in stage II, 2(3.6%) in stage III and 1(1.8%) in stage IV. The *p*-value was 0.519.

PR positive cases in stage I were 27(48.2%), 4(7.1%) in stage II, 5(8.9%) in stage III and 1(1.8%) in stage IV. The *p* value was 0.668. Three (5.4%) of Her-2 positive cases were in stage I and 1(1.8%) was in stage III. The *p* value was 0.774.

In conclusion statistically significant positive association was found for HER-2/neu expression with the histological types (*p*=0.002) as well as grades of the tumors (*p*=0.000) (Table-I). The association of HER-2/neu expression with the stage of tumor was statistically insignificant (*p*=0.519). No statistically significant association was found for histological type, grade and stage for ER (*p* =0.351, 0.538, 0.519) and PR (*p*=0 .604, 0.313, 0.668, respectively).

## DISCUSSION

This study was conducted at MH and CMH to find out the receptors′ status (ER, PR and Her-2/neu) in EC. In this study association of receptors status (ER, PR and Her-2/neu) was analyzed in correlation with the histological types, grades and stage of EC. In literature, ER and PR positivity is consistently seen in Type-I EC. It was found that 25 (44.6%) cases were positive for ER, 37 (66.1%) cases for PR and 4 (7.1%) were HER-2/neu positive.

Similar results have been seen in other studies also. In one study by Sirijaipracharoen et al., which included 108 patients, ER, PR and HER-2/neu expression were positive in 59.3%, 65.7% and 2.8% respectively.^14^

According to the results of this study, in low grade tumors (grade I and II), total ER positive cases were 20 (35.7%), PR positive cases were 30 (53.6%) and HER-2/neu were nil (0.0%). In grade III (high grade) tumors, ER positive cases were only 5 (8.9%), PR positive were 7 (12.5%) and HER-2 neu cases were 4 (7.1%). These findings are in concordance with previous studies. According to Sirijaipracharoen Sunamehok et al., ER positive cases in Grade I and II were 17 (36.2%), PR positive cases were 12 (21.44%) and HER-2/neu positive cases were 2 (4.3%).^14^

Studies have shown high grade EC has a high percentage of HER-2/neu overexpression (17%) and gene amplification (21%).^15^ This is consistent with the results of this study in which there were 12 high grade carcinomas out of which 4(33.3%) cases over expressed HER-2/neu by immunohistochemistry.

Positivity of HER-2/neu has been seen previously in high grade Endometrioid carcinoma and serous carcinoma.^16^ In a study by Slomovitz et al,^16^ 18% of Serous carcinomas over expressed HER-2/neu by immunohistochemistry. In the present study, out of three cases of Serous carcinoma, two were positive for HER-2/neu (66.6%). This percentage is much higher than that of above mentioned study. The reason for this may be attributed to the smaller sample size. Other studies have also shown higher rates of HER-2/neu overexpression by immunohistochemistry in high grade EC. Santin et al. found over expression of HER-2/neu in 80% of SC. Pratel et al., Berchuck et al., Khalifa et al. and Coronado et al found over expression of HER-2/neu in the order of 80%, 40%, 25% and 18% respectively.^16^-^19^

In the present study, histological types and grades of the tumors showed positive association with HER-2/neu expression, whereas no significant association of HER-2/neu was seen with the stage of EC.

Some earlier studies have considered HER-2/neu positivity and negativity without considering complete or incomplete staining^17^, while other studies were based only on the staining intensity of HER-2/neu.^17^ In this study, both percentage of complete and incomplete staining and the intensity of staining were taken according to ASCO/CAP guidelines.

## CONCLUSION

This study concludes that progesterone receptors (66.1%) are detected most frequently in EC cases followed by ER (44.6%) and Her-2/neu (7.1%) receptors. The associations of PR and ER with histological types, grades and stages were not significant. However, there is significant association of Her-2/neu receptors′ with high grade Endometroid and Serous carcinoma.

A significant proportion (75%) of high grade carcinomas has shown over expression of Her-2/neu with immunohistochemical analysis. High grade breast carcinoma that are Her-2/neu positive have shown better treatment and survival when treated with Herceptin. Since EC has shown Her-2/neu overexpression, it may be suggested that EC may also be treated on the same lines with anti Her-2/neu treatment for improvement of survival and reduction of morbidity. With such future perspectives for the overall management of the patients with EC, pathologists have to play an important role by analyzing immunohistochemistry results and helping in deciding the selection of the patients who can be treated by targeted therapy.

### Author`s Contribution

**Samina Waqar and Saleem Ahmad Khan** conceived, designed did statistical analysis and manuscript writing.

**Samina Waqar** did the data collection.

**Saba Waqar** did editing of manuscript.

**Saleem Ahmad Khan and Tariq Sarfraz** did review and final approval of manuscript.
